# Diabetic cardiomyopathy: prevalence, determinants and potential treatments

**DOI:** 10.1177/2042018819834869

**Published:** 2019-03-27

**Authors:** Gaurav S. Gulsin, Lavanya Athithan, Gerry P. McCann

**Affiliations:** Department of Cardiovascular Sciences, University of Leicester and the Leicester NIHR Biomedical Research Centre, Leicester, UK; Department of Cardiovascular Sciences, University of Leicester and the Leicester NIHR Biomedical Research Centre, Leicester, UK; Department of Cardiovascular Sciences, University of Leicester and the Leicester NIHR Biomedical Research Centre, Glenfield Hospital, Groby Road, Leicester LE3 9QP, UK

**Keywords:** cardiometabolic disease, diabetic cardiomyopathy, heart failure, type 2 diabetes

## Abstract

The prevalence of type 2 diabetes (T2D) has reached a pandemic scale. These patients are at a substantially elevated risk of developing cardiovascular disease, with heart failure (HF) being a leading cause of morbidity and mortality. Even in the absence of traditional risk factors, diabetes still confers up to a twofold increased risk of developing HF. This has led to identifying diabetes as an independent risk factor for HF and recognition of the distinct clinical entity, diabetic cardiomyopathy. Despite a wealth of research interest, the prevalence and determinants of diabetic cardiomyopathy remain uncertain. This limited understanding of the pathophysiology of diabetic heart disease has also hindered development of effective treatments. Tight blood-glucose and blood-pressure control have not convincingly been shown to reduce macrovascular outcomes in T2D. There is, however, emerging evidence that T2D is reversible and that the metabolic abnormalities can be reversed with weight loss. Increased aerobic exercise capacity is associated with significantly lower cardiovascular and overall mortality in diabetes. Whether such lifestyle modifications as weight loss and exercise may ameliorate the structural and functional derangements of the diabetic heart has yet to be established. In this review, the link between T2D and myocardial dysfunction is explored. Insights into the structural and functional perturbations that typify the diabetic heart are first described. This is followed by an examination of the pathophysiological mechanisms that contribute to the development of cardiovascular disease in T2D. Lastly, the current and emerging therapeutic strategies to prevent or ameliorate cardiac dysfunction in T2D are evaluated.

## Introduction

### The diabetes pandemic and cardiovascular disease

Diabetes mellitus (DM) is a major global health concern. Recent estimates suggest that there are currently 451 million people with diabetes worldwide and this figure is projected to increase to 693 million by 2045.^1^ Importantly, estimates suggest that almost half (49.7%) of people living with diabetes remain undiagnosed.^1^

The vast majority (90%) of people with diabetes have type 2 diabetes (T2D), which is linked to increased sedentary behaviour and obesity and is largely preventable. Whereas T2D was once rare in young people, increasingly we are seeing the condition diagnosed in children, adolescents and adults under the age of 30 years.^2,3^ Globally, there are now more obese than underweight people^4^ and this dramatic rise in obesity and sedentary lifestyles, particularly in younger age groups, has resulted in up to a 10-fold increase in the prevalence of T2D in younger adults.^5^

#### Diabetes mellitus and heart failure

The most deleterious consequence of developing T2D is a substantially elevated risk of cardiovascular disease (CVD). The risk of cardiovascular complications is two to two-and-a-half times greater in people with T2D compared with the nondiabetic population. In a meta-analysis combing data from 4,549,481 people with T2D, almost one third (32.7%) suffered from CVD and half of all deaths were attributable to CVD.^6^ Atherosclerotic diseases (angina, myocardial infarction and stroke) have typically been regarded as the predominant manifestations of CVD in T2D. However, recent data from the United Kingdom National Diabetes Audit 2015–2016, which includes data on over 2.7 million patients with diabetes, showed that heart failure (HF) is the commonest cardiovascular complication of T2D and a major cause of premature mortality.^7^ While the risk of death, myocardial infarction and stroke can be mitigated with strict risk-factor control in T2D, the excess risk of HF persists, in spite of good cardiovascular risk-factor management.^8^

Patients with DM have up to a 74% increased risk of developing HF, and diabetic patients with HF are four times more likely to die than those without HF.^9^ Importantly, unrecognized HF is highly prevalent in T2D, with over one quarter (27.7%) of over 60s having previously undiagnosed HF in one report.^10^ There is a high prevalence of diabetes in patients with both common forms of HF: impaired systolic left ventricular (LV) function and HF with preserved ejection fraction. Conversely, a high proportion (almost one third) of patients with DM have undiagnosed LV dysfunction.^10^

## Diabetic cardiomyopathy

Large, population-based studies have shown that the occurrence of HF in diabetes cannot be accounted for solely by the increased atherosclerotic risk^11–13^ or the prevalence of other traditional risk factors, such as age, sex, hypertension, coronary artery disease (CAD) and dyslipidaemia that are inherent in diabetic subjects. Even after adjustment for these factors, diabetes still confers a two- to five-fold added risk for HF development.^11–13^ This has led to the identification of diabetes as an independent risk factor for HF and the recognition of the distinct clinical entity of ‘diabetic cardiomyopathy’,^14^ a term originally coined by Lundbaek in 1954.^15^

Diabetic cardiomyopathy is defined as myocardial disease in patients with diabetes, not attributable to hypertension, CAD or other cardiac disease.^14^ Four stages of diabetic cardiomyopathy are described, and there is overlap with the HF classifications of both the American College of Cardiology/American Heart Association Stage and New York Heart Association Class (Table 1).^14^ Patients in stage 2 have annual mortality rates up to 20%^16^ and are twice as likely to be hospitalized for HF than nondiabetics with HF with preserved ejection fraction.^17^ Over the past 2 decades, the rapid evolution of advanced non-invasive cardiac imaging techniques has enabled detailed evaluation of heart structure and function *in vivo*. Application of these techniques to the study of diabetic cardiomyopathy have provided key insights to the relationship between T2D and HF.

**Table 1.** Classification of diabetic cardiomyopathy.

Diabetic cardiomyopathy stage	Stage 1	Stage 2	Stage 3	Stage 4
	Diastolic HF with normal ejection fraction	Symptomatic HF with combined systolic and diastolic dysfunction	Symptomatic HF to which hypertension, microvascular disease or viral disease have contributed No coronary artery disease	Symptomatic HF, with contribution from multiple confounders including coronary artery disease
NYHA functional class	Class 1	Class 2	Class 3	Class 4
	Asymptomatic, no limitation of physical activity	Slight limitation during ordinary physical activity, with fatigue, palpitation, dyspnoea or angina	Marked limitation, with symptoms occurring during minimal physical activity	Symptoms present at rest Unable to carry out any physical activity without discomfort
ACC/AHA HF stage	Stage A	Stage B	Stage C	Stage D
	At risk of HF, but no structural heart disease or symptoms	Asymptomatic structural heart disease	Symptomatic HF with structural heart disease	Refractory HF requiring specialist interventions

Classification of diabetic cardiomyopathy, using the New York Heart Association (NYHA) Functional Class and American College of Cardiology/American Heart Association (ACC/AHA) HF stages. There is considerable overlap across the three classification schemes.

HF, heart failure.

### Clinical factors associated with HF development in T2D

Specific to diabetes populations, increased age, increasing glycosylated haemoglobin (HbA1c), increased body mass index, hypertension, CAD, longer duration of diabetes and the presence of microvascular complications are associated with HF development.^18^ The UK National Diabetes Audit 2015–2016 showed that the association of HF with HbA1c level was relatively weak but considerably stronger for hypertension.^19^ Although females with T2D have a higher risk for cardiovascular complications than males,^20^ there are scarce data in relation to HF.^18^

### Morphological changes in the diabetic heart

The occurrence of structural changes of the diabetic myocardium were first observed by Rubler et al. in 1972. In four postmortem specimens from diabetic patients free from hypertension, CAD or valvular heart disease, Rubler and colleagues described findings of left ventricular (LV) hypertrophy and diffuse myocardial fibrosis.^21^ There is now an abundance of data to support Rubler and coworkers’ initial findings and demonstrate that diabetes is associated with several alterations in LV geometry.

#### Alterations in left-ventricular mass and volumes

Increased LV mass is independently associated with diabetes in many (but not all) echocardiographic^22–24^ and cardiovascular magnetic resonance imaging (CMR) studies.^25–27^ A 1% rise in HbA1c level was associated with a 3.0 g [95% confidence interval (CI) 1.5–4.6 g] increase in LV mass in one report.^23^ While these changes in LV mass are modest, increased LV mass is a recognized predictor of cardiovascular morbidity and mortality,^28,29^ and is likely to be a key contributor to HF development in T2D. When the increased LV mass is indexed for body surface area, however, the differences between diabetics and controls become inconsistent. These inconsistencies arise because adjustment of LV mass for body surface area inherently ‘permits’ obese individuals to have higher LV mass;^30^ this is why indexing of LV mass/height is advocated.^31,32^

Other markers of LV remodelling are also apparent in diabetes; LV mass/volume,^26,33^ relative wall thickness,^23,34–36^ and septal thickness^36,37^ are all increased in diabetes. LV mass may be increased as a consequence of increased ventricular wall thickness or from chamber dilatation, that is, the spectrum of LV hypertrophy ranges from concentric to eccentric hypertrophy. While there is variation in both the degree and pattern of hypertrophy observed in patients with diabetes, concentric LV hypertrophy represents the main structural characteristic of diabetic cardiomyopathy. Concentric LV remodelling is associated with an increased risk of developing HF and other adverse cardiac events and appears to be the predominant remodelling pattern in diabetes.^38,39^ However, LV geometry is also altered by sex,^40^ ethnicity,^41^ obesity^42^ and hypertension,^43^ common confounders in diabetes, and controlling for these confounders may in fact prevent the development of LV remodelling.^44^ The lack of standardization in reported markers of LV remodelling makes comparisons between studies difficult^45^ and limits knowledge of LV remodelling patterns in diabetes.

### Functional impairments in the diabetic heart

Cardiac dysfunction in diabetes is thought to lie on a continuum (Table 1), ranging from asymptomatic diastolic dysfunction through subclinical systolic dysfunction and then overt HF with reduced ejection fraction.^14^

#### Diastolic dysfunction

It is often stated that diastolic dysfunction is the earliest functional change occurring in diabetic cardiomyopathy. Observational studies have found an increased frequency of diastolic dysfunction in T2D by echo and CMR. The prevalence and severity of diastolic dysfunction was shown to be directly proportional to HbA1c level in one study of 1810 people with T2D.^46^ Despite controlling metabolic risk factors in T2D [e.g. elevated HbA1c, hypertension, raised body mass index (BMI), dyslipidaemia, albuminuria], diastolic dysfunction persists in the absence of LV remodelling or systolic impairment.^44^ There are, however, inconsistencies in the prevalence of diastolic dysfunction found in asymptomatic subjects with T2D. Reported prevalence rates vary from 15% to 78%^47–50^ and differ according to the technique used for diagnosis.^47^

#### Systolic dysfunction

Despite the association of T2D with HF, few studies have shown that T2D causes a reduction in global LV ejection fraction, which remains the most utilized measure of LV systolic performance. Using myocardial strain and strain-rate measurement, subclinical impairments in systolic function with normal ejection fraction are now frequently reported in T2D. Tissue Doppler imaging,^35,51,52^ speckle tracking echocardiography^23,53^ and CMR^33^ data confirm systolic LV global longitudinal strain is lower in T2D than in nondiabetics. These impairments in global longitudinal strain worsen with time^54^ and vary across the glycaemic spectrum (e.g. one study found global longitudinal strain in controls was −18.5 ± 2.3%; in subjects with prediabetes it was −18.1 ± 2.5% *versus* −17.8 ± 2.4% in those with T2D).^23^ These subclinical abnormalities in contractility are widely considered a precursor to the onset of clinical HF in diabetes. Indeed, longitudinal studies have found global longitudinal strain to be an independent predictor of cardiovascular events and may provide incremental prognostic value in asymptomatic people with T2D.^55,56^ However, in the first of these studies, the sample size was modest and there was significant risk of overfitting of the multivariable regression model,^55^ and in the second study, only echocardiographic parameters as predictors of outcomes were explored with no mention of clinical predictors.^56^ Importantly, the majority of cardiovascular events in these studies were atherosclerotic (myocardial infarction and stroke) and not HF-related events and it remains unclear why reductions in global longitudinal strain would predict atherothrombotic events independent of other clinical risk factors.

#### Combined systolic and diastolic dysfunction

The vast majority of studies that have examined both have shown that impaired systolic strain is associated with diastolic dysfunction.^23,36,50,52,53,57–60^ A small number have, however, reported reduced systolic strain without diastolic dysfunction.^33,61,62^ This could indicate that diastolic dysfunction is not the earliest functional change in the diabetic heart and is in fact preceded by impaired systolic strain. In most of these studies, diastolic function was determined by tissue Doppler velocities and not strain analyses, likely reducing the sensitivity of identifying diastolic impairments.^61,62^ Furthermore, it is acknowledged that different guidelines for grading diastolic dysfunction by echocardiography yield inconsistent results and may only be accurate for identifying the most severe cases.^63^ Assessment of diastolic strain rate may be a more sensitive measure of early diastolic impairment.^26^ Only one CMR study^33^ reported reduced LV systolic global longitudinal strain (controls −11.4 ± 2.8 *versus* T2D −9.6 ± 2.9, *p* = 0.049) and preserved diastolic strain rate (controls 0.65 ± 0.13 *versus* 0.62 ± 0.26 s^−1^, *p* = 0.749), but these values are much lower than those seen in the prevailing echo and CMR literature where they are typically ~20% and 1.5–2.0 s^−1^, respectively, in controls.

### Biomarkers in diabetic cardiomyopathy

Several studies have investigated the role of natriuretic peptides in asymptomatic patients with T2D.^64^ Serum N-terminal pro-B-type natriuretic peptide levels are higher in asymptomatic T2D with isolated diastolic dysfunction compared with controls.^65,66^ At present B-type natriuretic peptide is often used in routine practice, however natriuretic peptide levels can be influenced by many other factors, several of which are more prevalent in diabetes, including obesity, increasing age, use of renin–angiotensin–aldosterone-system inhibitors and renal dysfunction.^67^ And diabetes itself is associated with elevated natriuretic peptide levels.^67^ Numerous other biomarkers are being evaluated in HF^64^ and specifically, diabetic HF. In one study comparing biomarker profiles between diabetic and nondiabetic patients with HF with preserved ejection fraction, endothelin-1 (a potent vasoconstrictor), galectin-3 and carboxy-terminal telopeptide of collagen type 1 (profibrotic biomarkers) were higher in patients with diabetes.^17^ Of more interest, is a biomarker analysis of the ADVANCE trial, which assessed the prediction of new and worsening HF in a nested case-control analysis.^68^ This study demonstrated that measurement of N-terminal pro-B-type natriuretic peptide gave a similar predictive accuracy as clinical risk factors and was of additive value, although the effects were attenuated when patients with levels > 400 pg/ml (in the HF diagnostic range) were excluded. Markers of inflammation [interleukin 6, high-sensitivity C-reactive protein (IL-6, hsCRP)] and myocardial damage (troponin) were not of added value.^68^ The identification of plasma biomarkers in HF is an area of intense research interest and may impact on clinical management in the future.^64^

## Aetiological factors in the development of LV dysfunction

**Figure 1.**
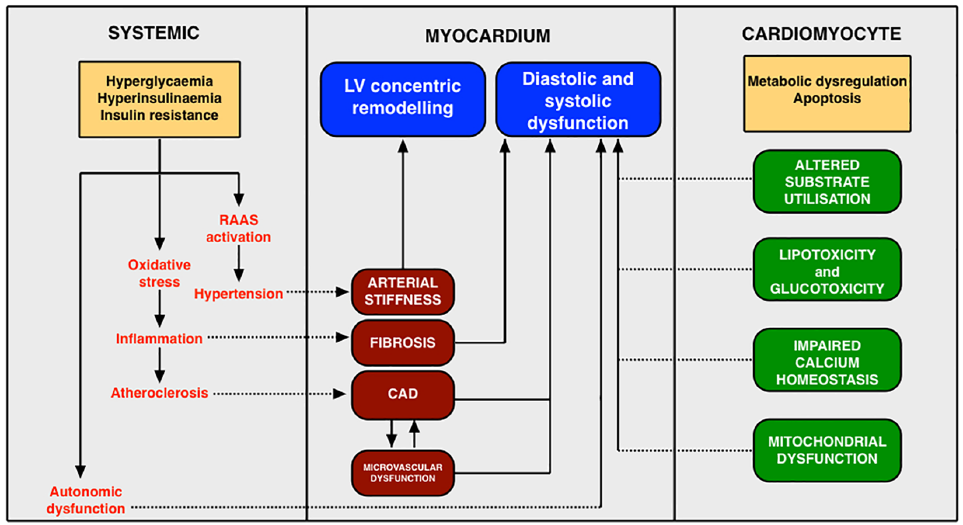
Local and systemic perturbations involved in the pathophysiology of diabetic cardiomyopathy. RAAS, renin–angiotensin–aldosterone system; CAD, coronary artery disease; LV, left ventricle.

The mechanisms contributing to the development of diabetic cardiomyopathy have been extensively explored in animal models.^69^ These include myocardial lipotoxicity and glucotoxicity, damage from glycated end products and reactive oxygen species, impaired calcium homeostasis, mitochondrial dysfunction, activation of the renin–angiotensin–aldosterone system, altered myocardial substrate utilization, and cardiac autonomic neuropathy (Figure 1).^70^

Several inverse correlations with diastolic dysfunction in T2D have been identified: HbA1c level,^57^ age,^48^ aortic stiffness,^26^ duration of DM,^26^ microvascular dysfunction^71^ and myocardial triglyceride content.^72–74^ Similarly, multiple associations with LV systolic strain are reported; these include BMI,^75^ waist circumference,^75^ HbA1c,^76^ blood pressure (BP),^60^ sex,^34^ presence of microalbuminuria,^34,77^ LV relative wall thickness,^34,77^ CAD^78^ and, again, myocardial triglyceride.^33^ Importantly, these findings were made on the basis of multivariate analyses, which have been limited by small sample sizes (seldom greater than 100 subjects) and incomplete datasets, with significant risk of overfitting the regression models.^79^

### Myocardial energy metabolism

Impaired myocardial substrate utilization and altered energy metabolism have recently been described in T2D and are likely to be contribute to the development of cardiac impairment. The normal heart derives 70% of its energy from free-fatty-acid metabolism and 30% from glucose metabolism.^80^ In T2D, there is a shift toward increased free-fatty-acid utilization by the myocardium in T2D due to increased free-fatty-acid availability. This is less energy efficient with lower adenosine triphosphate (ATP) yield^81^ and leads to metabolic inefficiency in the diabetic heart.^82^

Myocardial energetics can now be assessed non-invasively by phosphorus magnetic resonance spectroscopy. This enables assessment of the myocardial creatine phosphate (PCr)/ATP ratio, which is a sensitive index of the energetic state of the myocardium.^83^ Decreased myocardial PCr/ATP ratios have been demonstrated in T2D and suggest that myocardial energetic impairment is a key component in the pathophysiology of diabetic cardiomyopathy.^84–86^ Impairment in myocardial energetics has also been exacerbated by exercise^86^ and may reflect metabolic inflexibility in the diabetic heart. Importantly, a decreased PCr/ATP ratio has been linked to contractile dysfunction and is a predictor of mortality, although this was in patients with dilated cardiomyopathy.^87^

### Myocardial steatosis

**Figure 2.**
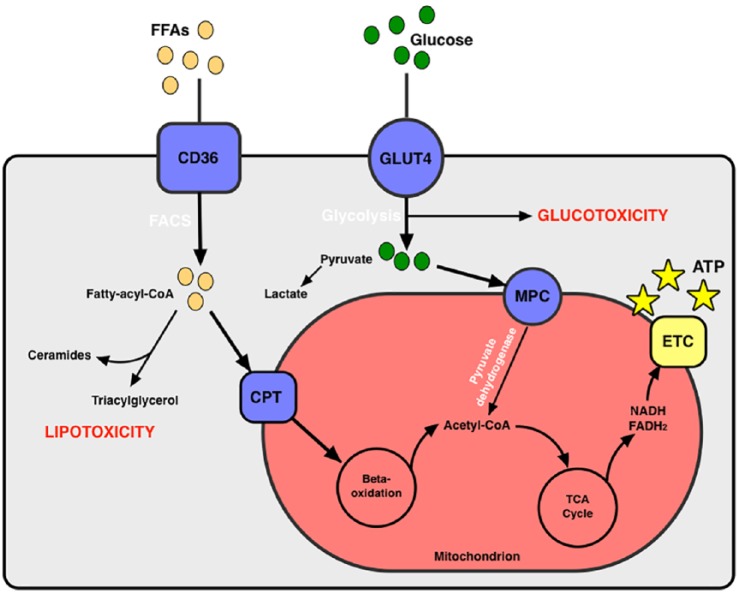
Myocyte energy metabolism and alterations that contribute to lipotoxicity and glucotoxicity. CoA, coenzyme A; CD36, cluster of differentiation 36; FACS, ; FFAs, free fatty acids; CPT, carnitine palmitoyltransferase; FADH_2_, flavine adenine dinucleotide; GLUT 4, glucose transporter type 4; MPC, mitochondrial pyruvate carrier; NADH, nicotinamide adenine dinucleotide; TCA, tricarboxylic acid; ETC, electron-transport chain; ATP, adenosine triphosphate.

Metabolic dysregulation is central to the pathogenesis of DM. Insulin resistance results in decreased availability of glucose in the myocardium.^88^ There is an increased supply of free fatty acids and a shift towards fatty-acid oxidation in the myocyte.^89^ The supply of fatty acids, however, exceeds the oxidative capacity of the heart and nonoxidative lipid metabolism ensues.^90^ Products of nonoxidative lipid metabolism include ceramide and diacylglycerol, which are toxic to cardiomyocytes and can induce myocardial dysfunction, apoptosis and fibrosis (Figure 2).^91^ Excessive myocardial triglyceride accumulation (steatosis) was first demonstrated in mouse models of DM^92^ and has emerged as a contributor to development of diabetic cardiomyopathy. Myocardial steatosis is mediated by microribonucleic acid (microRNA) dysregulation (such as miR-451 and miR-494-3p) and was linked with LV dysfunction in recent animal models of T2D and obesity,^93,94^ supporting a central role for microRNAs in the pathogenesis of diabetic cardiomyopathy.

Magnetic resonance spectroscopy also allows the detection of myocardial steatosis *in vivo*, and several studies have confirmed elevated myocardial triglyceride content in T2D (Table 2).^33,72,74,95^ Steatosis has been significantly correlated with diastolic strain rate on speckle tracking echocardiography^73^ and on CMR,^74^ and was also an independent predictor of systolic strain in a small study with 66 patients.^33^ However, all studies to date have involved small numbers. If confirmed in a larger sample size with appropriate adjustment for confounders, it will likely play a central mechanistic role in the pathophysiology of diabetic heart disease.

**Table 2.** Summary of magnetic resonance spectroscopy studies evaluating myocardial triglyceride content in T2D.

Study	Patients	*n*	Mean age (years)	M/F	Mean BMI (kg/m^2^)	Key outcomes
**McGavock** ^96^	Lean controls	15	35 ± 3	7/8	23 ± 2	Myocardial TG elevated in IGT and T2D *versus* controls (0.95 ± 0.60 *versus* 1.06 ± 0.62 *versus* 0.46 ± 0.30 fat/water, *p* < 0.05)
	Obese	21	36 ± 12	10/11	32 ± 5	
	IGT	20	49 ± 9	5/15	31 ± 6	
	T2D	78	47 ± 10	37/41	34 ± 7	
**Rijzewijk et al.** ^72^	Controls	28	54 ± 1	28/0	26.9 ± 0.5	Myocardial TG increased in T2D *versus* controls (0.96 ± 0.07% *versus* 0.65 ± 0.05%, *p* < 0.05)
	T2D	38	57 ± 1	38/0	28.1 ± 0.6	
**Korosoglou et al.** ^74^	Controls	16	62 ± 3	10/6	23.9 ± 2.5	Myocardial TG in T2D 0.86 ± 0.14 Association between TG and mean diastolic strain rate (*r* = −0.71, *p* < 0.001) and peak systolic strain rate (*r* = 0.41, *p* = 0.02)
	T2D	42	62 ± 6	26/16	31.6 ± 4.8	
**Levelt et al.** ^33^	Controls	20	54 ± 10	9/11	28.6 ± 2.8	Elevated myocardial TG in T2D (1.13 ± 0.78 *versus* 0.64 ± 0.52, *p* = 0.017) Negative correlation between TG and systolic strain (*r* = −0.40, *p* = 0.003)
	T2D	46	55 ± 9	24/22	29.6 ± 5.7	

BMI, body mass index; IGT, impaired glucose tolerance; TG, triglyceride; T2D, type 2 diabetes.

#### Coronary microvascular dysfunction

Abnormalities of vascular function are well described in T2D, including endothelial dysfunction. Impaired coronary microvascular function has been demonstrated in older,^25,35,74^ but not younger patients^26^ with T2D. The mechanisms contributing to coronary microvascular dysfunction include: endothelial dysfunction, increased myocardial mass with reduced capillary density, myocardial fibrosis and reduced transmyocardial perfusion gradient due to increased LV diastolic pressure.^97^ Impaired myocardial performance index, a marker of overall systolic and diastolic function, has also shown association with microvascular dysfunction in T2D.^98^ However, evidence of this relationship between microvascular dysfunction and diastolic impairment in T2D is conflicting. CMR studies have failed to identify an association,^26,74^ whereas an echo study using tissue Doppler indices found significant associations.^71^ However, none of these studies angiographically excluded epicardial CAD. The potential link between coronary microvascular dysfunction and diastolic impairment in T2D is, therefore, unclear. Moreover, microvascular dysfunction may coexist with and potentiate the effects of epicardial CAD on cardiac dysfunction.

#### Myocardial fibrosis

Myocardial fibrosis is thought to be mediated by damage from advanced glycation end products^99^ and apoptosis caused by lipotoxicity.^74^ CMR techniques now allow the non-invasive detection of replacement myocardial fibrosis (late gadolinium enhancement) and an estimate of diffuse interstitial fibrosis using T_1_ mapping and calculation of myocardial extracellular volume.^100^ Patients with diabetes have shorter global contrast-enhanced myocardial T_1_ times compared with controls, indicative of a higher burden of diffuse interstitial myocardial fibrosis.^101^ This is independently associated with myocardial systolic^101^ and diastolic function.^99,101^ Subjects with T2D have a higher extracellular volume fraction than controls,^102,103^ although the differences found are small (1–2%),^103^ and there is a large degree of overlap when control subjects are well matched to those with diabetes.^33^ Elevated extracellular volume fraction is associated with increased admissions for HF and mortality, but this was in patients already referred for a clinical CMR with inevitable selection bias.^103^

#### Arterial stiffness

The aorta is a conduit for the delivery of blood to peripheral tissues. In addition, the elastic properties of the aorta act to dampen the sudden fluctuations in BP generated by blood ejected from the LV during each cardiac cycle. This transforms the pulsatile stroke volume into continuous blood flow through the peripheral arterial tree.^104^ This buffering capacity of the thoracic aorta is essential for maintaining normal LV structure and function. Aortic stiffening is an increase in the elastic resistance of the aorta to deformation and naturally occurs with ageing but is additionally accelerated by the traditional cardiovascular risk factors.^105,106^ Increased aortic stiffness is a strong predictor of adverse cardiovascular events in several cohorts,^107–109^ including T2D.^110,111^ Our group has previously shown a modest but significant correlation exists between mean aortic distensibility and peak early diastolic strain rate (*r* = 0.564, *p =* 0.023) in young adults with T2D.^26^ This suggests that increased aortic stiffness is an early change in diabetes, which contributes to subclinical cardiac dysfunction in T2D, independent of age and BP.

Poorer blood-glucose control is associated with accelerated aortic stiffness, particularly in younger adults with T2D.^112^ Reducing HbA1c levels may attenuate the progression of aortic stiffness and some studies have shown that aortic stiffness is modifiable by diabetes treatment.^113–116^

Despite the recognition that aortic stiffening is a key determinant of LV dysfunction in several diseases, the precise mechanisms linking aortic stiffness with adverse cardiovascular outcomes are unclear. The most prominent hypothesis for the pathophysiological basis linking aortic stiffness with adverse cardiovascular events in T2D is the development of LV remodelling.^117^ Aortic stiffening disturbs the arterial–ventricular interaction, augmenting ventricular afterload and supplementing the development of LVH. This results in increased LV filling pressures and impairment in the passive flow of blood from the left atrium to the LV in early diastole. Aortic stiffness therefore likely mediates the development of diabetic cardiomyopathy by stimulating LVH. We have recently confirmed this in a cross-sectional study of 80 young adults with T2D, where we have demonstrated that aortic stiffness is an independent predictor of concentric LV remodelling.^118^ Given that aortic stiffening is potentially reversible with aggressive BP reduction,^119^ this may yield a potential therapeutic strategy for preventing or treating diabetic cardiomyopathy.

## Pharmacological interventions to reverse cardiovascular dysfunction in T2D

### Glycaemic control

The majority of previous, large, multicentre, randomized-controlled trials such as UKPDS,^120^ ADVANCE,^121^ ACCORD^122^ and VADT^123^ did not demonstrate an improvement in macrovascular outcomes with tight blood-glucose control. A meta-analysis of these four large trials, comprising over 27,000 patients assigned to more-intensive *versus* less-intensive blood-glucose control showed only modest reduction in major adverse cardiovascular events [hazard ratio (HR) 0.91; 95% CI 0.84–0.99].^124^ This was primarily driven by a reduction in myocardial infarction, with no overall benefit on all-cause or cardiovascular death.^124^ Similarly, a meta-analysis of data from eight randomized trials comprising 37,229 people with T2D revealed no observed benefit of intensive glycaemic control on HF-related outcomes.^125^ Even with intensive glucose control, underlying epigenetic alterations that promote oxidative stress, myocardial inflammation and LV dysfunction persisted in a mouse model of diabetes. This may be a key mechanism driving the persistence of cardiac dysfunction in the context of tight glycaemic control; and targeting epigenetic networks has been proposed as a novel strategy to ameliorate LV dysfunction in T2D.^126^

Recently, new classes of glucose-lowering therapies, such as glucagon-like peptide-1 (GLP-1) agonists,^127^ and inhibitors of sodium–glucose cotransporter 2 (SGLT2 inhibitors)^128^ have shown exciting results with improved glycaemic control, as well as reduced cardiovascular mortality in patients with T2D.^129^ However, these recent trials were designed to assess the safety and tolerability of these novel drugs, and therefore the mechanisms behind the observed cardiovascular benefits are speculative. Nevertheless, the increased use of these agents has been advocated in recent guidelines of management of hyperglycaemia in T2D.

### GLP-1 agonists

GLP-1 agonists exert their effects by suppressing appetite, glucagon secretion and gastric emptying, and by stimulating the release of insulin.^130^ In the randomized-controlled LEADER trial, patients with T2D and high cardiovascular risk treated with liraglutide had lower rates of cardiovascular death compared with those having placebo.^127^ Similarly, in high-risk T2D patients, cardiovascular event rates (death, nonfatal myocardial infarction and nonfatal stroke) were found to be significantly lower with semaglutide^131^ and albiglutide than for placebo.^132^ However, in the EXSCEL trial of the GLP-1, exenatide *versus* placebo, there was no overall cardiovascular risk benefit with the study drug, although this study included patients with or without a prior history of CVD^133^ (Table 3). In the PIONEER-6 study, the cardiovascular safety and efficacy of the first oral formulation of a GLP-1 agonist (semaglutide) will be compared with placebo in over 3000 high-risk patients with T2D.^134^ Although the complete results of this trial are yet to published, early data indicate a 51% relative risk reduction in cardiovascular death and a 49% relative risk reduction in all-cause mortality with semaglutide, but no difference in the risk of nonfatal myocardial infarction or stroke compared with placebo.^135^ Treatment with semaglutide was also associated with a 13.8% reduction in weight in obese people in a phase II clinical trial.^136^ Notably, the beneficial effects of GLP-1 agonists appear to be on atherosclerotic CVD, rather than lowering the risk of HF development. Agents with costimulatory effects on GLP-1 and glucose-dependent insulinotropic polypeptide receptors have recently emerged, which have demonstrated superior glucose and weight-lowering properties than GLP-1 analogues alone.^137^ The cardiovascular benefits of these drugs have yet to be demonstrated.

**Table 3.** Cardiovascular outcome trials of sodium–glucose cotransporter-2 inhibitors and glucagon-like peptide–receptor analogues.

Study	Agent	Sample size, *n*	Key inclusion criteria	Mean age, years	Median follow-up duration, years	Key findings
Sodium–glucose-cotransporter-2 inhibitors						
EMPA-REG OUTCOME^138^	Empagliflozin	Total: 7020 Drug: 4687 Placebo: 2333	T2D and CVD, HbA1c 7–10%	63.2	3.1	14% ↓ in primary outcome, 38% ↓ CV death, 13% ↓ MI, 24% ↑ stroke, 35% ↓ heart-failure hospitalization
CANVAS^139^	Canagliflozin	Total: 10,142 Drug: 5795 Placebo: 4347	T2D and history of or high risk for CVD, HbA1c 7–10.5%	63.3	2.4	14% ↓ in primary outcome, 13% ↓ CV death, 15% ↓ MI, 10% ↓ stroke, 33% ↓ heart-failure hospitalization
DECLARE-TIMI 58^140^	Dapagliflozin	Total: 17160 Drug: 8582 Placebo: 8578	T2D with and without history of CVD, HbA1c	64.0	4.2	7% ↓ in primary outcome, 17% ↓ CV death, 11% ↓ MI, 27% ↓ heart-failure hospitalization
Glucagon-like peptide–receptor analogues						
LEADER^127^	Liraglutide	Total: 9340 Drug: 4668 Placebo: 4672	T2D and CVD, HbA1c ⩾ 7.0%	64.3	3.8	13% ↓ in primary outcome, 22% ↓ CV death, 12% ↓ MI, 11% ↓ stroke, 13% ↓ heart-failure hospitalization
SUSTAIN-6^131^	Semaglutide	Total: 3297 Drug: 1648 Placebo: 1649	T2D and CVD, HbA1c ⩾7.0%	64.5	2.1	26% ↓ in primary outcome, 2% ↓ CV death, 26% ↓ MI, 39% ↓ stroke, 11% ↑ heart-failure hospitalization
EXSCEL^133^	Exenatide	Total: 14752 Drug: 7356 Placebo: 7396	T2D, 70% with CVD and 30% without, HbA1c 6.5–10%	62.0	3.2	Non-inferior but not superior to placebo for primary outcome No significant difference in rates of CV death, MI, stroke or heart-failure hospitalization between groups
HARMONY OUTCOMES^132^	Albiglutide	Total: 9463 Drug: 4731 Placebo: 4732	T2D and CVD, HbA1c >7%	64.1	1.5	22% ↓ in primary outcome, 7% ↓ CV death, 25% ↓ MI, 14% ↓ stroke

CV, cardiovascular; CVD, cardiovascular disease; HbA1c, glycosylated haemoglobin; MI, myocardial infarction; T2D, type 2 diabetes.

### SGLT2 inhibitors

SGLT2 inhibitors have emerged as glucose-lowering therapies showing improved cardiovascular outcomes in T2D (Table 3). In the first of these, the EMPA-REG OUTCOME^138^ and CANVAS^139^ studies, there was a relative risk reduction in cardiovascular mortality and hospitalization for HF in patients with T2D and established, or at high risk of, CVD. More recently, in the largest of the SGLT2 inhibitor trials with the longest follow-up duration, the DECLARE-TIMI 58, a study of the SGLT2 inhibitor dapagliflozin *versus* placebo, reduced rates of hospitalization for HF were also observed in lower-risk subjects with T2D.^140^ Furthermore, when participants of the EMPA-REG OUTCOME were stratified according to risk of HF development at baseline, the beneficial effects of empagliflozin on reducing incident HF-related events were observed in patients at low, intermediate and high risk of HF.^141^ These latter studies suggest a potential role for SGLT2 inhibitors in the prevention of HF development in low-risk patients with T2D.

SGLT2 inhibitors lower blood-glucose levels by promoting urinary glucose excretion. Secondary effects include weight loss, a modest diuretic effect and BP reduction.^142^ The precise mechanisms linking SGLT2 inhibitors to lower risk of HF and favourable cardiovascular outcomes are unclear. The most popular hypothesis is that the increased fluid losses (driven by urinary glucose and sodium excretion) lead to a reduction in intravascular volume and systolic BP. This in turn reduces preload and afterload, leading to improvements in myocardial oxygen supply and vascular function.^142^ A mediation analysis of the EMPA-REG OUTCOME trial has indeed demonstrated that changes in plasma volume are central to the CV risk benefits observed with empagliflozin.^143^ Furthermore, in a randomized trial of empagliflozin *versus* placebo in patients with T2D and uncontrolled nocturnal hypertension, empagliflozin was associated with significant 24 h ambulatory BP reductions compared with placebo (−10.0 *versus* −2.4 mmHg, respectively, over 12 weeks, *p* < 0.001).^144^ Others suggest that SGLT2 inhibitors, through a shift in myocardial metabolism towards ketones, have favourable effects on cardiac energetics.^145^ The results of these studies, while promising, should be viewed with a degree of caution. HF risk reduction was not the primary endpoint of either study and was based on investigator-reported HF events rather than objective measures (such as echocardiography or measurement of B-type natriuretic peptide levels). Nevertheless, several mechanistic studies are now underway to identify the specific cardiovascular effects of SGLT2 inhibitors. Whether the same cardiovascular benefits of SGLT2 inhibitors are seen in earlier stages of diabetic cardiomyopathy remains to be established.

### Blood pressure reduction

Patients with diabetes are twice as likely to suffer from hypertension than nondiabetics.^146^ Coexistence of these two conditions confers a greater risk of CVD, including CAD, LV hypertrophy, stroke and HF, compared with either diabetes or hypertension in isolation.^147^ Despite the high prevalence of hypertension in diabetes, there are inconsistencies in the recommended BP targets in these patients.^148–150^ Previous recommendations favoured an intensive approach to BP management in diabetes given the high CVD risk profile of these patients. Indeed, in nondiabetic patients at high risk of CVD, intensive BP lowering (to a systolic BP < 120 mmHg) dramatically lowers cardiovascular risk and all-cause mortality.^151^ It has therefore been suggested that tighter BP control be targeted in patients with T2D,^152^ although there are limited data to support this strategy as a means of overall cardiovascular risk reduction.

In the ACCORD BP study, which specifically addressed the issue of intensive BP lowering in T2D, there was no demonstrable survival benefit with intensive BP reduction (systolic BP < 120 mmHg) compared with a standard BP reduction target (<140 mmHg) over a median follow-up period of 4.7 years in 4733 patients with T2D.^153^ The annual incidence of all-cause mortality was similar with either BP target (1.28 and 1.19%, respectively; HR 1.07; 95% CI 0.85–1.35, *p =* 0.52). Intensive BP treatment was in fact harmful and led to increased incidence of syncope and hyperkalaemia.^154^ Even after longer-term follow up (median duration 8.8 years) was carried out for 3957 patients from the ACCORD study, there remained no reduction in the rate of a composite of fatal and nonfatal major cardiovascular events or mortality with intensive *versus* standard BP control.^154^ Similarly, patients with T2D and a history of CAD do not appear to benefit from an intensive BP-lowering-treatment strategy.^155^ Recent data suggest a U-shaped relationship between BP and cardiovascular outcomes in T2D, where systolic BP over 150 mmHg or less than 110 mmHg portends a poorer prognosis.^156^ Maintenance of BP within this range appears to be the most appropriate strategy in T2D. In view of these and other large prospective studies evaluating BP-lowering targets in DM, current National Institute for Health and Care Excellence (NG28) recommendations are that BP be maintained below 140/80 mmHg in uncomplicated T2D or below 130/80 mmHg if there is a history of kidney, eye or cerebrovascular disease.^148^

### Lifestyle interventions to reverse cardiovascular dysfunction

#### Weight loss

T2D has long been regarded as a chronic condition capable of being ameliorated but not cured. However, proof that T2D is a reversible condition has been firmly established in patients undergoing bariatric surgery^157,158^ and in a primary-care-led administration of a 825–853 kcal/day meal-replacement diet.^159^ The extent of weight loss is strongly linked to reversal of T2D. Insulin use, diabetes duration and high HbA1c levels reduce the chances of reversal.^158^ None of these reports, however, have assessed changes in cardiovascular function.

In obese subjects without T2D, sustained weight loss, either with diet or after surgery, has resulted in favourable reductions in CMR-measured LV mass, volumes, arterial stiffness and diastolic function.^160^ Improved diastolic function following weight loss in obesity has been associated with improved energetics^161^ and with reduced myocardial triglyceride content.^162^

In patients with insulin-treated T2D, a 471 kcal/day very-low-energy diet has also been shown to reduce myocardial steatosis (0.88 ± 0.12% to 0.64 ± 0.14%, *p* = 0.02) in a small (*n* = 12) single-group study and was associated with improved diastolic filling on CMR.^163^ Interestingly, a recent brief report from the same group, suggests that in the first few days after commencing a very-low-energy diet, there may actually be an increase in steatosis and reduced diastolic mitral filling.^164^ However, it should be noted that there was a dramatic decrease in ventricular volumes, with a nonsignificant decrease in estimated filling pressure, which are both likely to have affected the diastolic filling rate.

Recently, the selective-serotonin-2C-receptor agonist lorcaserin, which suppresses appetite, has been shown to cause sustained weight loss, reduce hyperglycaemia and reduce the risk of microvascular complications in high-risk overweight and obese patients with and without diabetes.^165,166^ Whether this leads to lower macrovascular complications remains to be demonstrated. Alter-natively Lorcarserin may be used as an adjunct to surgical or dietary strategies as a means to supplement or maintain weight reduction.

#### Exercise programmes

Large cohort studies have shown that increased aerobic exercise capacity is associated with significantly lower cardiovascular and overall mortality in men^167^ and women^168^ with DM. Peak exercise capacity (maximal volume of oxygen uptake) is a recognized prognostic marker in subjects with CVD^169^ and in T2D.^170^ In a small study (*n* = 19), exercise capacity was significantly reduced in subjects with T2D having diastolic dysfunction compared with those having normal diastolic function.^171^ This suggests that improvements in exercise capacity may yield improvements in cardiac dysfunction in T2D; at the very least, in the early stages of diabetic cardiomyopathy. This is supported by a recent position paper from the European Association of Preventive Cardiology, which advocates the promotion of individualized exercise training programmes in people with T2D to improve both cardiovascular and metabolic function.^172^

Intervention studies have demonstrated a strong causal link between exercise training and glycaemic control in those with T2D. Exercise training has consistently been found to lower HbA1c by 0.6–0.7%, with greater effects seen with higher volumes of exercise.^173,174^ Importantly, these substantial benefits are maintained when exercise training does not result in weight loss.^173,174^ This is consistent with experimental studies that have elucidated key insulin-dependent and insulin-independent pathways linking physical activity to improved glucose regulation that do not act through adiposity.^175,176^

While the benefits of exercise training on glycaemic control are well established, the effects on diastolic function are less well known, primarily due to insufficient data, differences in measurement and poor study design. However, encouraging data are starting to emerge in those with obesity and chronic disease. For example, an 8-week exercise training programme in obese men improved diastolic function to levels seen in lean controls, despite no weight loss.^177^ Similarly, just 4 weeks of exercise training has been shown to improve diastolic function in those with HF with similar improvements also observed in a matched cohort without HF.^178^

Only two studies have been conducted in T2D assessing the effects of exercise training on diastolic function. In a small pilot study, 3 months of aerobic exercise training reversed diastolic dysfunction in almost half (45%) of individuals with T2D and grade 1 diastolic dysfunction,^179^ while another study found no overall effect.^180^ However in the latter study, a *post hoc* analysis revealed that change in moderate-intensity physical activity was significantly associated with change in myocardial strain rate, although it is unclear whether this was systolic, diastolic or both.^180^ This mirrors the wider evidence where light-to-moderate aerobic exercise training has repeatedly been demonstrated to improve diastolic function across a number of groups.^177,179^ The effectiveness of vigorous-intensity exercise or combined aerobic and resistance training is less well established, with at least one study showing the latter approach is not effective.^181^ Given this evidence base, it is important the efficacy of aerobic exercise training is investigated further.

Finally, in the randomized-controlled LookAHEAD trial, the effects of intensive-lifestyle intervention (which included a combined dietary weight loss and exercise programme) *versus* a diabetes support programme were evaluated in 5145 overweight or obese (mean age 58.7 years, BMI 36 kg/m^2^) people with T2D over a median follow-up duration of 9.6 years. Disappointingly, there was no difference in the rate of cardiovascular events in the intensive-lifestyle-intervention arm, despite a greater extent of weight loss, increased fitness and improved glycaemic control.^182^ However, the mean weight loss achieved in the intensive-lifestyle-intervention arm was only 6% by the end of the trial, and both study groups had intensive medical management of cardiovascular risk factors, which may have limited the treatment effect in the intervention arm.

## Conclusions

The rapid increase in prevalence of T2D now represents a global pandemic. These patients are at high risk of developing HF and dying prematurely, but the prevalence of subclinical cardiac dysfunction and the causes are uncertain. Improved glycaemic control *per se* does not reduce the risk of developing HF, but newer pharmacologic agents reduce CV complications and SGLT2 inhibitors have been shown to decrease HF-related hospitalizations. Weight loss, either with low-calorie diets or bariatric surgery, is also an attractive option for reversing diabetes and the risk of HF, but further studies are needed.
